# Interval debulking surgery with or without hyperthermic intraperitoneal chemotherapy in advanced-stage ovarian cancer: Single-institution cohort study

**DOI:** 10.3389/fonc.2022.936099

**Published:** 2022-07-28

**Authors:** Yong Jae Lee, Ki Eun Seon, Dae Chul Jung, Jung-Yun Lee, Eun Ji Nam, Sang Wun Kim, Sunghoon Kim, Young Tae Kim

**Affiliations:** ^1^ Department of Obstetrics and Gynecology, Institute of Women’s Life Medical Science, Yonsei University College of Medicine, Seoul, South Korea; ^2^ Department of Radiology, Yonsei Severance Hospital, Research Institute of Radiological Science, Yonsei University College of Medicine, Seoul, South Korea

**Keywords:** ovarian cancer, interval debulking surgery (IDS), hyperthermic intraperitoneal chemotherapy (hipec), neoadjuvant chemotherapy (NAC), Advanced stage ovarian cancer

## Abstract

To evaluate the additive effects of hyperthermic intraperitoneal chemotherapy (HIPEC) to interval debulking surgery (IDS) in patients with advanced-stage ovarian cancer. From January 2015 to February 2019, 123 patients with stages IIIC-IV ovarian cancer were treated with neoadjuvant chemotherapy (NAC) followed by IDS with optimal cytoreduction. Forty-three patients received IDS with HIPEC and 80 patients had IDS without HIPEC. The median follow-up period was 34.4 months. No differences in baseline characteristics in patients were found between the two groups. The IDS with HIPEC group had fewer median cycles of chemotherapy (*P* = 0.002) than the IDS group. The IDS with HIPEC group had a higher rate of high surgical complexity score (*P* = 0.032) and higher rate of complete resection (*P* = 0.041) compared to the IDS group. The times to start adjuvant chemotherapy were longer in the IDS with HIPEC group compared to the IDS group (*P* < 0.001). Postoperative grade 3 or 4 complications were similar in the two groups (*P* = 0.237). Kaplan-Meier analysis showed that HIPEC with the IDS group had better progression-free survival (PFS) (*P* = 0.010), while there was no difference in overall survival between the two groups (*P* = 0.142). In the multivariate analysis, HIPEC was significantly associated with better PFS (HR, 0.60; 95% CI, 0.39 - 0.93). The addition of HIPEC to IDS resulted in longer PFS than IDS without HIPEC not affecting the safety profile. Further research is needed to evaluate the true place of HIPEC in the era of targeted treatments.

## Introduction

Ovarian cancer is one of the most lethal gynecological malignancies in women  (1). Maximal cytoreductive surgery combined with platinum-based chemotherapy is the standard treatment for advanced epithelial ovarian cancer  (2). However, even after complete remission after primary treatment, approximately 60%-80% of patients with advanced-stage disease experience relapse  (3). To improve survival outcomes, several new combinations with platinum-based chemotherapy have been investigated, including target agent, antiangiogenic agent, immunotherapy, and intraperitoneal chemotherapy  (4).

Hyperthermic intraperitoneal chemotherapy (HIPEC) combines intraperitoneal chemotherapy with hyperthermia. It maintains a high chemotherapeutic drug concentration in tumor cells and reduces the side effects of intraperitoneal catheter-related complications. In addition, hyperthermia enhances the penetration of chemotherapy agents at the peritoneal surface, and increases the cytotoxic effect of chemotherapy  (5). Recently, Van Driel et al.  (6) showed significant survival benefits for stage III ovarian cancer patients treated with HIPEC followed by interval debulking surgery (IDS) compared to surgery alone and it is not associated with higher rates of side effects.

Despite the existence of randomized phase III trials using HIPEC at IDS in advanced-stage ovarian cancer, its widespread adoption is not high in real-world practice. There are difficulties in the incorporation of HIPEC in IDS  (7). Patients undergoing IDS with HIPEC have longer surgery time than those who perform IDS alone, and use high-temperature chemotherapy agents, which may increase the incidence of perioperative complications. Adding HIPEC to IDS may increase the hospital length of stay. We have applied an institutional HIPEC program in the management of advanced-stage ovarian cancer since 2015  (8). The aim of this study is to evaluate the outcomes of the addition of HIPEC to IDS in patients with advanced-stage ovarian cancer.

## Materials and methods

### Study populations

**Figure 1:**
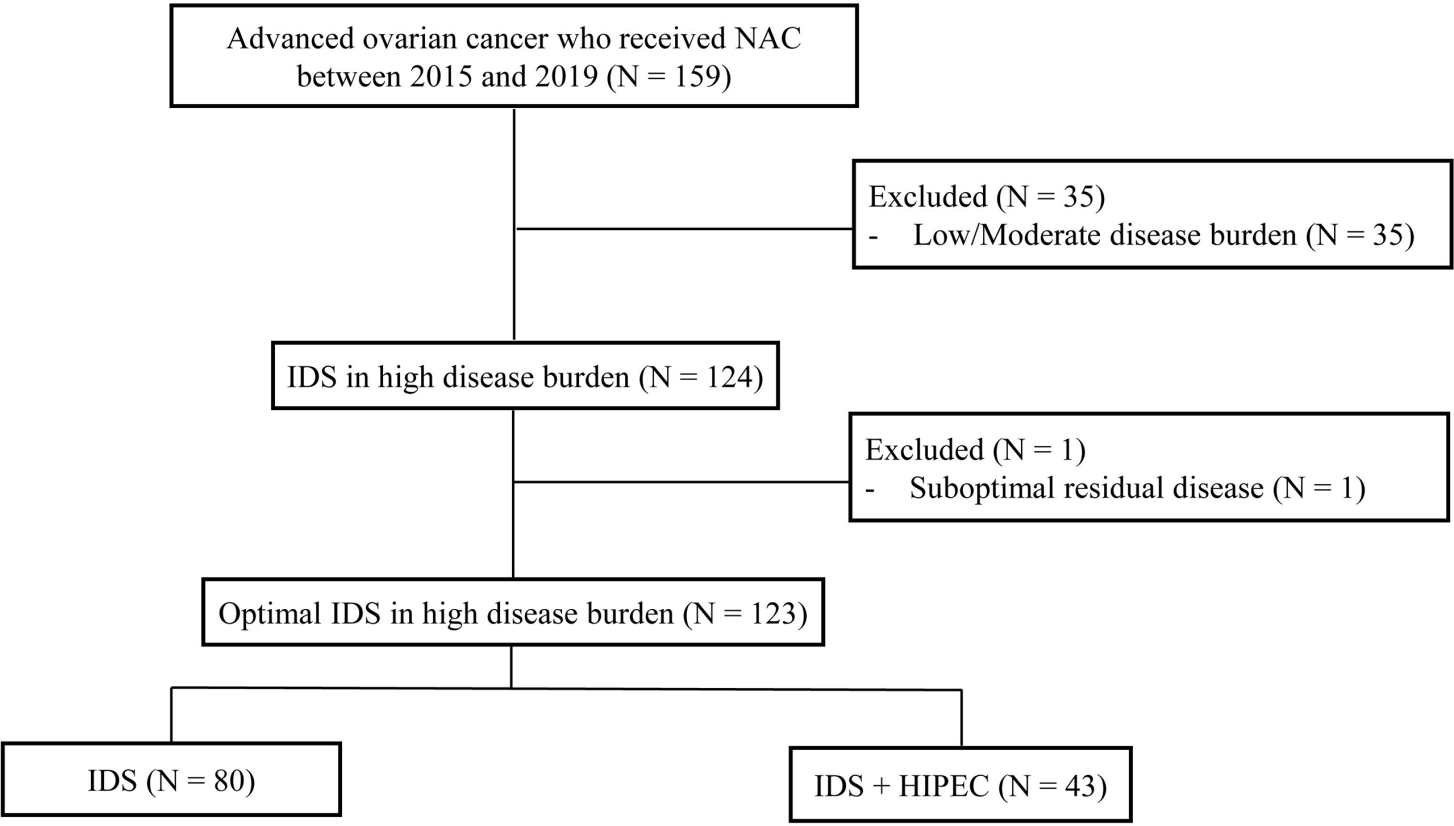
Flow diagram of the study population. NAC, neoadjuvant chemotherapy.

We retrospectively reviewed the medical records of 159 patients with pathologically confirmed ovarian cancer who received neoadjuvant chemotherapy (NAC) from 2015 and 2019 at the Yonsei Cancer Center, Seoul, South Korea. The incorporation of HIPEC with IDS was first performed at Yonsei Cancer Hospital in 2015. Inclusion criteria were as follows (1): histopathologically confirmed International Federation of Gynecology and Obstetrics stage III or IV ovarian, fallopian tube, and primary peritoneal carcinoma. In the cases of stage IV, the extra-abdominal disease was mostly supradiaphragmatic lymph node metastasis found on chest computed tomography (CT) or positron-emission tomography-computed tomography (PET-CT) before NAC. Most patients achieved near-complete remission (no uptake on PET-CT, no gross lesions on chest CT) of distant metastasis after NAC. When the distant metastatic lesion remained, it was removed from the IDS (2) patients who underwent IDS after NAC (3) patients who received more than one cycle of NAC before IDS. We excluded patients who had low/moderate tumor burden assessed by CT at the time of diagnosis (n = 35) and patients who underwent suboptimal surgery at the time of IDS (n = 1). After this review, the final study population comprised 123 patients (**Figure 1**). This study was reviewed and approved by the institutional review board (IRB) at Severance Hospital, Yonsei University Health System, Seoul, Korea (IRB number: 4-2021-0451).

### Advanced-stage ovarian cancer treatment

All the patients were evaluated in order to determine the tumor burden of ovarian cancer. The diagnostic baseline workup included esophagogastroduodenoscopy, colonoscopy, and computed tomography (CT) of the chest, abdomen, pelvis, and positron-emission tomography CT (PET-CT).

Our institution applied the following selection criteria for the use of NAC as the primary treatment strategy. NAC was performed on the basis of patient performance status and medical comorbidities, radiologic imaging (abdominopelvic or chest CT, PET-CT), and diagnostic laparoscopy  (8). These patients were further classified according to tumor burden. The extent of tumor was used to develop the preoperative disease score (DS). The tumor burden was classified as: DS low: pelvic and retroperitoneal spread; DS moderate: additional spread to the abdomen without upper abdomen disease; DS high: the presence of upper abdominal tumor affecting the diaphragm, spleen, liver, or pancreas  (9). All radiologic imaging was reviewed by an experienced gynecologic radiologist.

All patients, preferably, were recommended to receive three cycles of NAC, IDS with or without HIPEC, and three cycles of postoperative adjuvant chemotherapy (POAC). Total cycles of NAC or POAC were determined at the clinician’s discretion. For NAC and POAC, all patients received platinum-based chemotherapy and none of the patients received Poly (ADP-ribose) polymerase inhibitors. All patients underwent surgery with the intent to remove all visible tumors and to achieve complete cytoreduction (R0). The complexity of surgical procedures was classified as low (surgical complexity score (SCS) 1 to 3), intermediate (SCS 4 to 7), or high (SCS ≥ 8)  (10, 11). Each surgical procedure was assigned a score from 1 to 3 according to its complexity. The surgical procedures scored as 1 are hysterectomy, bilateral salpingo-oophorectomy, omentectomy, pelvic lymphadenectomy, para-aortic lymphadenectomy, abdominal peritoneum stripping, and small bowel resection. Scored as 2 in complexity are large bowel resections, diaphragm stripping/resections, splenectomy, and liver resections. Recto-sigmoidectomy with anastomosis are scored as 3, the most complex.

HIPEC was performed immediately after IDS. Of 43 HIPEC cases, nine were performed using laparoscopic technique and 34 were performed using the closed technique. Cisplatin (100 mg/m^2^, n = 17) or paclitaxel (175 mg/m^2^, n = 26) were used, and chemotherapy agents were diluted in 3 L of 1.5% dextrose solution for peritoneal dialysis. Initially, 3 L of a heated perfusion solution was infused into the abdominal cavity at a rate of 800-1000 mL/min through the inflow tube using the Belmont Hyperthermic Pump (Belmont Instrument Corporation, Billerica, MA, USA). Three intra-abdominal thermometers (one positioned in the pelvis and two in the diaphragm area) were used to monitor the temperature inside the peritoneal cavity during the infusion, which remained a constant 42°C. The duration of the HIPEC procedure was 90 minutes, after which the perfusion solution was completely drained, and bowel anastomosis was performed if needed. To prevent nephrotoxicity in patients who underwent HIPEC with cisplatin, an intravenous bolus (9 g per square meter in 200 ml) was administered at the start of HIPEC perfusion, followed by a continuous infusion (12 g per square meter in 1000 ml) over 6 hours

### Endpoints and statistical analysis

The primary endpoint was progression-free survival (PFS) and the secondary endpoints included perioperative morbidity and overall survival (OS). Disease progression was evaluated with the response evaluation criteria in solid tumors, version 1.1.  (12) PFS was defined as the time from the date of diagnosis to disease progression and OS was defined as the time from diagnosis until death due to any cause. Perioperative complications (within 30 days postoperatively) were graded according to the Memorial Sloan-Kettering Cancer Center surgical secondary events grading system  (13). Other adverse events were graded according to the National Cancer Institute Common Terminology Criteria for Adverse Events Version 5.0.

Demographic data are summarized as the median (range) or frequency (percentage). Chi-square and Fisher exact tests were used to compare variables. PFS and OS curves were estimated using the Kaplan-Meier method. Cox regression analysis was used to investigate the effects of the prognostic factors, expressed as hazard ratios (HRs) with 95% confidence intervals (CIs). The analyses were performed using SPSS (version 21.0; IBM Corp., Armonk, NY, USA). Statistical significance was assumed at P < 0.05.

## Results

### Patients’ characteristics

The clinical characteristics of patients are shown in **Table 1**. Patients were categorized according to IDS and IDS with HIPEC. A total of 123 patients were included in this study. Forty-three patients (35.0%) received HIPEC, and 80 patients (65.0%) did not receive HIPEC. There were no differences in median age, histologic type, FIGO stage, ASA score, BRCA mutation status, median CA-125 level, and median estimated blood loss between the two groups (IDS or IDS with HIPEC). The IDS with HIPEC group had fewer median cycles of chemotherapy (6 vs. 9, P = 0.002).

**Table 1:** Baseline patients’ characteristics.

Variable	IDS (n = 80)	IDS + HIPEC (n = 43)	*P*
Median age, years (range)	60 (27-78)	60 (40-76)	0.590
Histologic type, n (%)			0.183
High grade serous	76 (95.0%)	39 (90.7%)	
Other^a^	4 (5.0%)	4 (9.3%)	
FIGO stage, n (%)			
III	38 (47.6%)	16 (37.2%)	0.065
IV	42 (52.6%)	27 (62.8%)	
ASA score before NAC, n (%)			0.255
1	3 (3.8%)	1 (2.3%)	
2	37 (46.1%)	25 (58.2%)	
3	40 (50.1)	17 (39.5%)	
BRCA, n (%)			0.054
Wild-type	47 (58.8%)	31 (72.1%)	
BRCA1/2 mutation	20 (25.0%)	11 (25.6%)	
Not applicable	13 (16.2%)	1 (2.3%)	
Median CA-125 level, U/mL (range)	1514.8 (94.2-17911.3)	1961.4 (108.6-17303.1)	0.686
Total cycles of chemotherapy, median (range)	9 (4 - 12)	6 (6 - 9)	0.002

IDS, Interval Debulking Surgery; FIGO, International Federation of Gynecology and Obstetrics; NAC, neoadjuvant chemotherapy; ASA, American Society of Anesthesiologists;

a Clear cell, mucinous, endometrioid, carcinosarcoma, squamous cell, seromucinous.

### Surgical outcomes

The surgical characteristics of patients are shown in **Table 2**. There was a significantly higher rate of high SCS (42.9% vs. 27.5%, P = 0.032). In the IDS group, the rate of intermediate SCS was higher than that in the IDS with HIPEC group. The IDS with HIPEC group had a significantly higher rate of R0 (62.8% vs. 47.5%, P = 0.041) compared to the IDS group. The IDS with HIPEC group had significantly longer mean operative time (486.0 vs. 289.5 min, P < 0.001) because of the additional 90 minutes of HIPEC perfusion and massive irrigation of the abdominal cavity. Median time interval between surgery and the start of adjuvant chemotherapy was significantly longer in the IDS with HIPEC group compared to the IDS group (21 vs. 16 days, P < 0.001).

**Table 2:** Surgical characteristics.

Variable	IDS (n = 80)	IDS + HIPEC (n = 43)	*P*
Surgical complexity score			0.032
Low (≤3)	8 (10.0%)	8 (19.0%)	
Intermediate (4-7)	50 (62.5%)	16 (38.1%)	
High (≥8)	22 (27.5%)	18 (42.9%)	
Residual disease, n (%)			0.041
No gross tumor	38 (47.5%)	27 (62.8%)	
≤0.5 cm	29 (36.3%)	15 (34.9%)	
>0.5 cm and ≤1.0 cm	13 (16.2%)	1 (2.3%)	
Estimated blood loss, median (range), ml	425 (20-7000)	700 (50-3600)	0.101
Mean operative time, min	289.5 (100-840)	486.0 (251-915)	<0.001
Median time interval between surgery and the start of adjuvant chemotherapy	16 (7-50)	21 (13-41)	<0.001

### Safety and treatment administration

Comparison of postoperative complications are shown in **Table 3**. Most of the grade 2 complications that occurred after surgery were patients who underwent transfusion with anemia and most patients were corrected within 3 days of transfusion. In patients with grade 2 complication, patients treated with intravenous and oral medications for postoperative pulmonary thromboembolism received the longest duration of treatment. The most common grade 3 adverse events in the IDS with HIPEC group were pleural effusion (14%), hydronephrosis (2.3%), and breast hematoma (2.3%). The patients with breast hematoma complication underwent total mastectomy with axillary lymph node dissection because of breast cancer during IDS with HIPEC and underwent emergency surgery for postoperative breast hematoma. In the IDS group, the most common grade 3 adverse events were pleural effusion (6.3%), pelvic fluid collection (1.3%), anastomotic leakage (1.3%), and pneumonia (1.3%). No patients died within 30 days postoperatively (grade V events). There was no significant difference in postoperative grade 3 or 4 complications between the two group (P = 0.237).

**Table 3:** Postoperative complications according to the Memorial Sloan-Kettering Cancer Center surgical secondary events grading system from days 0 to 30.

	IDS (n = 80)	IDS + HIPEC (n = 43)	*P*
Complication grade, n (%)^a^			0.283
No	28 (35.0%)	11 (25.6%)	
1	6 (7.5%)	6 (14.0%)	
2	38 (47.5%)	18 (41.9%)	
3	8 (10.0%)	8 (18.6%)	
Grade 3,4 complications, n (%)			0.237
Pleural effusion	5 (6.3%)	6 (14.0%)	
Pelvic fluid collection	1 (1.3%)	0 (0%)	
Anastomotic leakage	1 (1.3%)	0 (0%)	
Hydronephrosis	0 (0%)	1 (2.3%)	
Pneumonia	1 (1.3%)	0 (0%)	
Breast hematoma	0 (0%)	1 (2.3%)	

a The highest grade of complication per patient was considered (if there was more than 1).

### Survival outcomes

**Figure 2:**
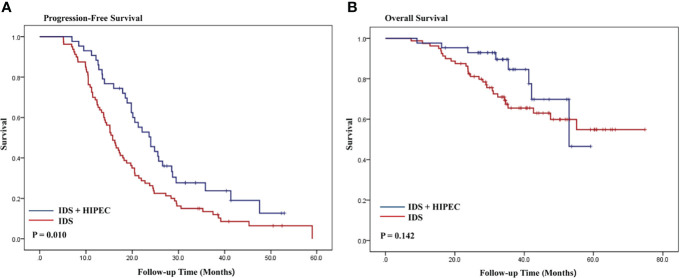
Kaplan-Meier curves of progression-free survival and overall survival according to HIPEC in patients with high disease burden **(A, B)**. IDS, interval debulking surgery.

The Kaplan–Meier curves for OS and PFS are shown in **Figure 2**. The median follow-up was 34.4 months in patients with high disease burden. The median PFS was 23.6 and 15.8 months for IDS with HIPEC and IDS, respectively. The median OS was 33.9 and 34.5 months for IDS with HIPEC and IDS, respectively. Patient who underwent IDS with HIPEC significantly prolonged the PFS (P = 0.010), while the difference in OS was not significant (P = 0.142). In patients who underwent HIPEC, there was no significant difference in the survival outcome between the paclitaxel and cisplatin groups (**Supplementary File 1**).

The results of the multivariate Cox regression analyses of PFS and OS in patients with high disease burden are shown in **Table 4**. The multivariate analysis showed that HIPEC was an independent prognostic factor associated with a lower risk of progression (HR, 0.60; 95% CI, 0.39 - 0.93), while HIPEC was not an independent prognostic factor for death (HR, 0.93; 95% CI, 0.33 - 2.64). We adjusted the following variables for multivariate analysis: age, ASA score, CA-125 level, FIGO stage, histology, residual disease after IDS, estimated blood loss, operative time, time interval between surgery and the start of adjuvant chemotherapy, SCS, BRCA mutation, and cycles of total chemotherapy.

**Table 4:** Multivariate analyses for progression-free and overall survival using a Cox proportional hazards model with categorical variables.

Variables	PFS		OS	
	HR (95% CI)	*P*	HR (95% CI)	*P*
HIPEC				
IDS	1.00		1.00	
IDS + HIPEC	0.60 (0.39-0.93)	0.023	0.93 (0.33-2.64)	0.896

ASA, American Society of Anesthesiologists; CI, confidence interval; FIGO, International Federation of Gynecology and Obstetrics; IDS, interval debulking surgery; HR, hazard ratio; PFS, progression-free survival; OS, overall survival.

Models were adjusted by age, ASA score, CA-125 level, FIGO stage, histology, residual disease after IDS, estimated blood loss, operative time, time interval between surgery and the start of adjuvant chemotherapy, chemotherapy regimen, surgical complexity score, BRCA mutation, and cycles of total chemotherapy.

## Discussion

To the best of our knowledge, this is the first real-world experience showing survival outcomes after publication of the OVHIPEC trial. In this study, we evaluated the impact of the incorporation of HIPEC at the time of IDS on survival outcomes in patients with advanced-stage ovarian cancer. Our study indicates that HIPEC with complete or optimal IDS resulted in longer PFS than IDS alone. Furthermore, we showed the feasibility and acceptable side effects of performing HIPEC in IDS for patients with advanced-stage ovarian cancer.

Several retrospective studies have shown that IDS with HIPEC for the treatment of advanced-stage ovarian cancer is feasible and reasonably well tolerated  (14–16). Van Driel et al.  (6) presented a phase III randomized clinical trial of HIPEC with IDS for stage III epithelial ovarian cancer. In this study, patients treated with HIPEC with IDS after NAC had improved survival outcomes among stage III epithelial ovarian cancer patients. The implementation of HIPEC resulted in a longer recurrence-free survival and OS compared to IDS alone and resulted in acceptable perioperative morbidity. Ghirardi et al.  (17) analyzed the feasibility and perioperative morbidity in a single center. In their study, the incorporation of HIPEC to IDS is feasible and does not lead to an increased perioperative complication.

However, there are still controversies on whether IDS with HIPEC can be considered as a standard of treatment for advanced-stage ovarian cancer. Chiva et al.  (18) reported that the use of HIPEC for advanced and for recurrent ovarian cancer does not show any advantage of survival outcomes in a systemic review of 22 publications with 1450 patients. Jou et al.  (19) showed that the ovarian cancer patients treated with IDS with HIPEC had a higher risk for platinum refractory or resistant disease (50% vs. 23%; RR=2.18; 95% CI 1.11, 4.30, p=0.024). IDS with HIPEC was associated with higher grade 3 or 4 postoperative complications (65% vs. 4%) without improving survival outcomes. Tsip et al.  (20) reported the short-term results of HIPEC in advanced-stage ovarian cancer patients. HIPEC was associated with negative effects on metabolism and a longer period of restoration of the liver functions, which resulted in delaying the initiation of adjuvant chemotherapy. Lim et al.  (21) reported the preliminary results for a randomized multicenter trial of HIPEC in primary advanced epithelial ovarian cancer at the American Society of Clinical Oncology annual meeting in 2017. One hundred eighty-four patients with stage III and IV were randomly enrolled to HIPEC arm or no HIPEC arm after an optimal upfront surgery or IDS. There was no significant difference in survival outcomes between the two arms. In the subgroup analysis of NAC, the survival curves showed a trend toward gradual distinction favoring HIPEC. The authors reported that more long-term follow-up is required to confirm the impact of HIPEC in NAC groups.

The best timing of HIPEC seems to be at IDS after NAC in ovarian cancer. Applying HIPEC during IDS can lead to uninterrupted chemotherapy between NAC and adjuvant chemotherapy. Patients with favorable response to NAC are more likely to require less aggressive surgery at the time of IDS than primary debulking surgery. Third, IDS with HIPEC involves the direct delivery of a hyperthermic cytotoxic agent to the peritoneal surfaces and could be effective on minimal residual tumors after NAC. However, there are many unanswered questions in relation to IDS with HIPEC. Recurrence patterns and subsequent survival outcomes are unknown in patients treated with IDS with HIPEC. Chambers et al.  (22) showed that HIPEC may alter the pattern of disease recurrences in ovarian cancer patients. In advanced or recurrent ovarian cancer patients treated with cytoreductive surgery with HIPEC, the majority of patients experienced extra-peritoneal recurrences. Sinukumar et al.  (23) reported that systemic recurrences were common in advanced-stage ovarian cancer patient treated with IDS with HIPEC following NAC. These findings suggest that IDS with HIPEC may improve the intraperitoneal disease control within the abdomen due to the treatment of a hyperthermic cytotoxic agent to the peritoneal surfaces but may not contribute to control extra-peritoneal or systemic recurrences. There is no consensus on the role of HIPEC in the era of novel targeted therapies. Several studies showed that hyperthermia has been shown to degrade BRCA2, and produced the impairment of DNA repair in tumor cells with increasing the sensitivity to platinum chemotherapy  (24, 25). Whether the survival benefit of HIPEC in patients with BRCA mutation and the survival benefit of targeted agents after implementation of IDS with HIPEC remains to be known. Further studies are needed to evaluate the true place of HIPEC in the current era of targeted treatment.

The strength of our study is that we also included patients with stage IV at diagnosis who showed a complete remission at distant metastatic sites or resectable distant tumor at the time of IDS. It might be a rationale for adding HIPEC in IDS for stage IV to improve PFS. The OVHIPEC trial included only stage III disease, which may lead to selection bias. We diagnose more stage IV disease with preoperative imaging than in the past. With stage IV patients are excluded, at least one-third of advanced-stage ovarian cancer might be excluded from the study. The second unique aspect with our study is the real-world data of patients with similar clinical characteristics at the same institution and within the same timeframe who did not receive HIPEC. The OVHIPEC trial had the long recruitment period, which resulted in a small number of patients enrolled per year. In addition, there was no information on the quality of participating centers and clinicians, which could have affected the post-operative complication rate and survival outcome. Third, we included patients with a high tumor burden which was classified according to the extent of tumor at the time of diagnosis. Our study had some limitations. First, the small sample sized and retrospective study. Second, the median follow-up was only 34.4 months; the short follow-up period makes it difficult to draw any conclusions on overall survival. Third, the different types of drugs (cisplatin, paclitaxel) used in HIPEC may result in bias in data interpretation.

In conclusion, our results show that patients diagnosed with a high tumor burden and who achieved optimal resection in IDS after NAC had significantly better PFS with HIPEC during IDS as compared with IDS without HIPEC. Furthermore, the incorporation of IDS followed by HIPEC seems to be feasible and safe for the treatment of advanced-stage ovarian cancer patients. Additional larger randomized trials are needed to determine if IDS with HIPEC has survival benefits as a first-line treatment and to select the patients who will benefit the most from IDS with HIPEC. Further study is needed to evaluate the role of HIPEC in the current era of new and effective target agents.

## Data availability statement

The raw data supporting the conclusions of this article will be made available by the authors without undue reservation.

## Ethics statement

The studies involving human participants were reviewed and approved by This study was reviewed and approved by the institutional review board (IRB) at Severance Hospital, Yonsei University Health System, Seoul, Korea (IRB number: 4-2021-0451). The patient records were anonymized and de-identified before analysis. The IRB approved our study with a waiver of informed consent because this study involved no risk to the patients and no interventions. Written informed consent for participation was not required for this study in accordance with the national legislation and the institutional requirements.

## Author contributions

Conceptualization, YL and J-YL, Methodology, EN. Validation, SWK, Formal analysis, SHK, Investigation, SHK, Resources, YL, Data curation, YL. and DJ, Writing—original draft preparation, YL, Writing—review and editing, J-YL and YK, Supervision, J-YL. All authors contributed to the article and approved the submitted version.

## Funding

This study was supported by the grant of Industry-Academic Cooperation Foundation, Yonsei University from Shin Poong Pharmaceutical Co., LTd (2021-31-1352). The funder was not involved in the study design, collection, analysis, interpretation of data, the writing of this article or the decision to submit it for publication.

## Conflict of interest

The authors declare that the study was conducted in the absence of any commercial or financial relationships that could be construed as a potential conflict of interest.

## Publisher’s note

All claims expressed in this article are solely those of the authors and do not necessarily represent those of their affiliated organizations, or those of the publisher, the editors and the reviewers. Any product that may be evaluated in this article, or claim that may be made by its manufacturer, is not guaranteed or endorsed by the publisher.
