# Supplementary figures and images for: Interval debulking surgery with or without hyperthermic intraperitoneal chemotherapy in advanced-stage ovarian cancer: Single-institution cohort study
Source: Front Oncol. 2022 Jul 28;12:936099. doi: 10.3389/fonc.2022.936099 (PMC9367968; doi:10.3389/fonc.2022.936099)

**A Progression-Free Survival**

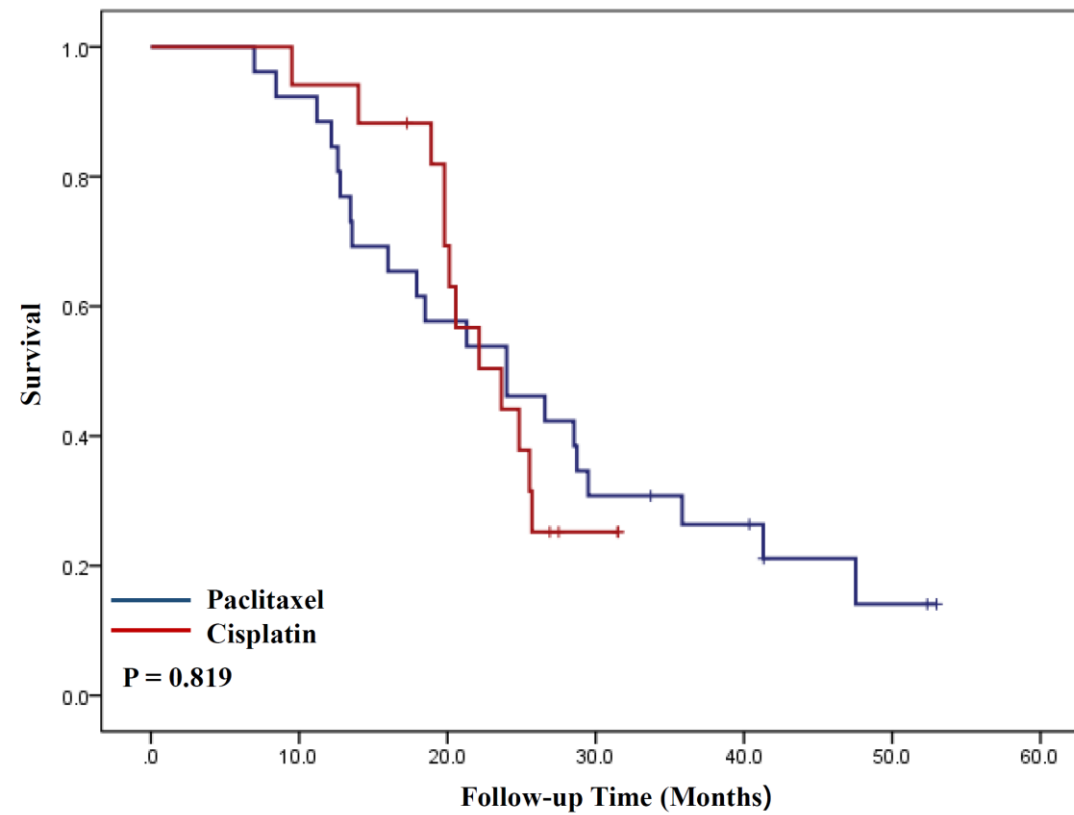

Supplement: Supplementary file 1 [file DataSheet_1.pdf]
